# Next-Generation Light Harvesting: MXene (Ti_3_C_2_T_x_)-Based Metamaterial Absorbers for a Broad Wavelength Range from 0.3 μm to 18 μm

**DOI:** 10.3390/ma18143273

**Published:** 2025-07-11

**Authors:** Abida Parveen, Deepika Tyagi, Vijay Laxmi, Naeem Ullah, Faisal Ahmad, Ahsan Irshad, Keyu Tao, Zhengbiao Ouyang

**Affiliations:** 1 Key Laboratory of Optoelectronic Devices and Systems of Ministry of Education and Guangdong Province, College of Physics and Optoelectronic Engineering, Shenzhen University, Shenzhen 518060, China; 2 THz Technology Laboratory, Shenzhen Key Laboratory of Micro-Nano Photonic Information Technology, Shenzhen University, Shenzhen 518060, China; 3 College of Mechatronics and Control Engineering, Shenzhen University, Shenzhen 518000, China; 4 College of Physics and Optoelectronic Engineering, Shenzhen University, Shenzhen 518060, China; 5 Shenzhen Key Laboratory for Nano-Biosensing Technology, Research Center for Biosensor and Nanotheranostic, School of Biomedical Engineering, Shenzhen University Medical School, Shenzhen University, Shenzhen 518060, China; 6 Department of Physics, University of Poonch Rawalakot, Rawalakot 12350, Pakistan

**Keywords:** Mxene, metamaterial absorbers, light harvesting

## Abstract

Electromagnetic wave (EMW) absorption materials are crucial for a wide range of applications, yet most existing materials suffer from complex fabrication and narrow absorption bands, particularly under harsh environmental conditions. In this study, we introduce a broadband metamaterial absorber based on Ti_3_C_2_O_2_ MXene, a novel two-dimensional material that uniquely combines high electrical and metallic conductivity with hydrophilicity, biocompatibility, and an extensive surface area. Through advanced finite-difference time-domain (FDTD) simulations, the proposed absorber achieves over 95% absorption from 0.3 µm to 18 µm. Additionally, other MXene variants, including Ti_3_C_2_F_2_ and Ti_3_C_2_(OH)_2_, demonstrate robust absorption above 85%. This absorber not only outperforms previously reported structures in terms of efficiency and spectral coverage but also opens avenues for integration into applications such as infrared sensing, energy harvesting, wearable electronics, and Internet of Things (IoT) systems.

## 1. Introduction

With the rapid advancement of wireless communication and the deployment of 5G technology, the issue of increasing managing electromagnetic wave (EMW) energy across broad spectral domains has become a critical design challenge [1,2,3]. To address this, researchers are exploring high-efficiency EMW absorption materials that combine broad bandwidth, lightweight properties, thin profiles, and strong absorption capacities [4]. Traditional EMW absorption materials typically incorporate a combination of electric and magnetic loss components, utilizing materials such as carbon-based materials (such as carbon nanotubes, carbon fibers, and graphene) and magnetic materials like cobalt, iron, and nickel [5,6]. While these materials excel in specific applications, they often fall short in extreme environments that require properties like light weight, durability, and corrosion-resistance.

In recent years, the two-dimensional (2D) materials, particularly transition metal carbides known as MXenes (e.g., Ti_3_C_2_T_x_) [7], have emerged as promising candidates for EMW absorption. Mxenes offer remarkable structural and chemical properties, including super high conductivity and abundant surface functional groups, which have attracted much attention in the field [8]. The unique 2D structure extends the wave propagation path of electromagnetic waves, which enhances the absorption [9,10]. However, the high conductivity of MXenes can also result in excessive EMW reflection, limiting their practical efficiency in certain applications. MXenes are a family of two-dimensional (2D) materials derived from the selective etching of “A” layers from MAX phases, where M is an early transition metal, A is an A-group element, and X is carbon and/or nitrogen. Their general formula is M_n+1_X_n_T_x_ (n = 1–4), where T_x_ represents surface terminations such as −O, −OH, and −F introduced during synthesis. MXenes exist in various morphologies, including monolayers, multilayers, and scroll-like structures, each offering distinct electromagnetic and optical properties. Surface functionalization significantly influences their conductivity, stability, and plasmonic behavior, thus enabling tunable performance in light harvesting, sensing, and energy conversion applications. This structural flexibility, combined with their large surface area and high metallic conductivity, makes MXenes highly suitable for broadband metamaterial absorber designs [11,12]. Several experimental studies support the practical viability of MXene-based absorbers. For example, Li et al. [13] demonstrated the broadband infrared transparency of Ti_3_C_2_T_x_ MXenes, while another study experimentally demonstrated that Ti_3_C_2_ MXene composites with in situ grown carbon nanotubes significantly enhance electromagnetic wave absorption [14].

Metamaterials, which are artificially engineered composites with properties not found in natural materials, have garnered attention for their ability to manipulate electromagnetic waves in ways that traditional materials cannot do. By adjusting the dimensions and shape of the unit cells, metamaterials can achieve desired permittivity or permeability, making them suitable for a variety of applications, including light trapping, imaging [15,16,17], and sensing [18,19,20], Among these, metamaterial perfect absorbers (MPAs) have gained particular interest for their ability to absorb electromagnetic radiation across broad frequency ranges, with applications in energy harvesting, thermal emission, and other energy-related technologies. MPAs are typically composed of metal and dielectric layers arranged in a metal/insulator/metal (MIM) configuration, offering dynamic control of absorbed energy and exhibiting sub-wavelength thicknesses, which are crucial for many scientific and technological applications [21,22,23,24,25,26,27].

Recent developments in MXene-based metamaterial absorbers have demonstrated great potential in addressing these challenges of EMW absorption. MXenes, synthesized by etching MAX phases (transition metal carbides or nitrides), possess remarkable properties including high specific surface area, mechanical strength, conductivity, and lightweight nature. These characteristics make MXenes ideal candidates for integration into metamaterial structures designed for broad-spectrum EMW absorption. Notably, Ti_3_C_2_T_x_ MXenes have shown excellent performance in electromagnetic interference shielding and EMW absorption due to their high dielectric loss and the ability to facilitate multiple reflections within their layered structure, leading to enhanced absorption [14,28,29,30].

To further improve the absorption performance of MXene-based metamaterials, recent research has focused on combining MXenes with other materials to enhance both dielectric and magnetic losses, which are crucial for boosting the overall performance of EMW absorbers. The addition of metals like gold or silver, or other conductive materials such as titanium nitride (TiN), has been explored to enhance the broad absorption capabilities of MXene-based metamaterials. However, despite these advancements, challenges remain in terms of production cost, absorption bandwidth, energy loss, and complex design configurations, which hinder the widespread application of these absorbers. Despite the promising properties of MXenes, no prior study has demonstrated a broadband Ti_3_C_2_O_2_-based metamaterial absorber extending from the visible to far-infrared (0.3–18 μm) with >95% absorption.

Here, we propose a new approach using Ti_3_C_2_O_2_-based MXene metamaterial absorbers, designed to operate efficiently across a broad range of wavelengths from 0.3 µm to 18 µm. Through finite-difference time-domain (FDTD) simulations, we investigate the impact of various structural modifications on the absorber’s performance. The Ti_3_C_2_O_2_ MXene-based absorber achieves over 95% absorption across this spectrum, while alternative MXene configurations, such as Ti_3_C_2_F_2_ and Ti_3_C_2_(OH)_2_, maintain absorption rates above 85%. The exceptionally broad absorption bandwidth from 0.3 to 18 μm arises from the synergistic interplay of several physical mechanisms unique to the MXene–Al_2_O_3_ design. First, Ti_3_C_2_T_x_ MXene’s inherent metallic and lossy dielectric properties enable plasmonic excitation and intrinsic impedance matching over a wide frequency range. Second, the absorber’s performance is strongly influenced by the thickness of the MXene layer, which governs field confinement and absorption tuning without requiring geometric resonators. Finally, the use of a non-patterned, planar architecture supported by a low-index Al_2_O_3_ substrate ensures non-resonant broadband absorption, in contrast to narrowband behavior typically seen in resonator-based or multilayer absorbers. This minimalist yet effective approach distinguishes our work from prior studies using graphene, MDM stacks, or TMO composites that rely heavily on structural complexity or external biasing.

This work aims to bridge the gaps in current MXene-based absorbers by offering a cost-effective, highly efficient, and scalable solution that can be applied in diverse fields such as infrared sensing, energy harvesting, and wearable technology. The results of this study contribute valuable insights into the design, optimization, and potential applications of MXene-based metamaterials in energy, photonics, and other advanced technologies.

## 2. Simulation Structure and Theory

### 2.1. Absorbers Design

**Figure 1:**
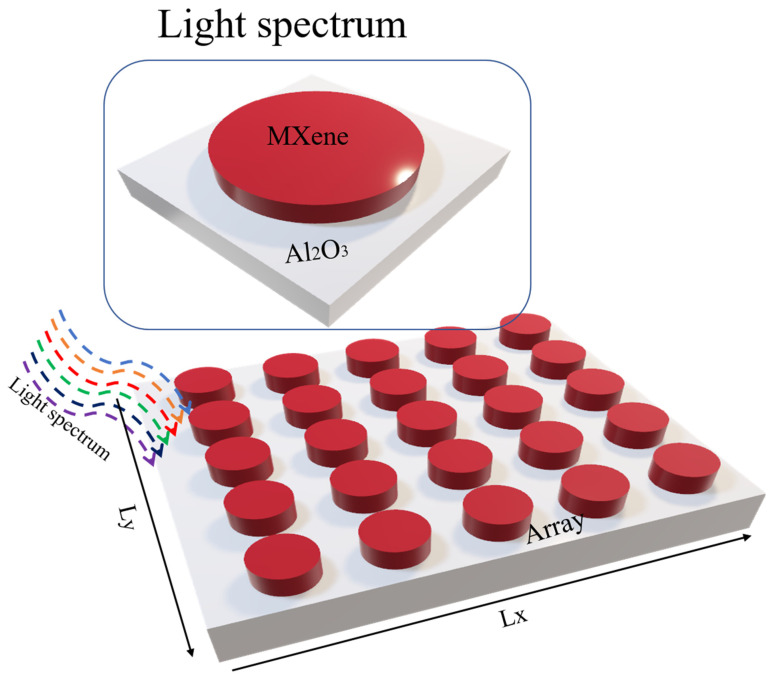
Schematic representation of the 3D MXene-based metamaterial absorber structure, with Lx = 5 μm, Ly = 5 μm, h_Al2O3_ = 1 μm, and h_Mxene_ = 8 μm.

The MXene layer was modeled with a thickness of 8 µm, while the substrate (Al_2_O_3_) was set to 1 µm shown in Figure 1. The dielectric constant of Al_2_O_3_ was taken from the Palik database, and the optical properties of Ti_3_C_2_O_2_ were based on experimentally reported values, accounting for both real and imaginary parts of the refractive index. The proposed model uses a multilayer MXene structure, typical in experimental films made by vacuum deposition or spin-coating. Optical constants are based on Ti_3_C_2_O_2_ multilayers [13], with an 8 μm thickness reflecting practical fabrication. Compared to monolayers, multilayers offer better conductivity and mechanical stability for absorber applications. The structure was simulated using the finite-difference time-domain (FDTD) method, a well-established numerical approach for solving Maxwell’s equations in the time domain. A unit cell model was constructed with periodic boundary conditions in the x- and y-directions, and perfectly matched layers (PMLs) were applied along the z-direction. A non-uniform mesh was used, with a fine mesh override region of 0.1 nm × 0.1 nm × 0.1 nm specifically defined around the MXene layer and near the Al_2_O_3_ interface to accurately capture near-field interactions. The remaining simulation domain used an automatically generated mesh optimized for computational efficiency. The simulation time step was determined by the Courant stability condition. The model was illuminated with a normally incident plane wave, and the simulations covered a broad wavelength range from 0.3 µm to 18 µm, encompassing the visible, near-infrared (NIR), and mid- to long-wave infrared (MIR–LWIR) regions. The period length of the unit structure was set as px = py = 5 μm. To estimate simulation reliability, we performed a mesh independence test, with cell sizes ranging from 2 to 5 nm. Absorptivity variation was found to be less than ±3%, indicating acceptable convergence. The interface effects in the layered MXenes, such as surface termination irregularities and oxidation, may cause real-world deviations, which will be addressed in future experimental studies.

### 2.2. Theory and Simulation Modeling

The absorption of electromagnetic waves in a material is governed by the Beer–Lambert law, which relates the material’s absorption coefficient *α*, thickness *d*, intensity of the incident light *I*_0_, and transmitted light *I* through the equation:

(1) $$I=I_{0}\times e^{-\alpha \cdot d}$$

where *I* is the intensity of the transmitted light, and the absorption coefficient *α* is given by $\alpha =\frac{4\pi k}{\lambda }$. Here, k is known as the extinction coefficient, which varies depending on the frequency of the incident light and the material’s intrinsic properties.

In this study, the absorption characteristics of the MXene layer are influenced by its thickness, with the MXene-Al_2_O_3_ combination facilitating resonant modes that enhance absorption. When incident light strikes the MXene layer, surface plasmon resonances are excited at the metal-insulator interface, generating destructive interference that traps light within the dielectric cavity, significantly boosting absorption. The variation in layer thickness influences these resonances through Fabry–Perot cavity effects, where constructive interference at specific thicknesses leads to enhanced light confinement and absorption.

Both thickness and material density significantly influence the optical response of metamaterial absorbers. Thickness affects the optical path, Fabry–Perot resonances, and impedance matching, while density impacts permittivity and losses through structural compactness. Our simulations use experimentally validated refractive indices of Ti_3_C_2_O_2_ MXene, reflecting typical density-related behavior. However, fabrication-induced density variations may affect absorption, which future experimental work should further investigate. Moreover, the thickness of the bottom metallic ground plane exceeds the penetration depth of the incident wave, transmission is nearly eliminated, leading to perfect absorption. This relationship between layer thicknesses of layers and the absorption underscores the importance of careful design and optimization to maximize absorption efficiency and minimize unwanted reflections in metamaterial absorbers:

(2) $$A=1-R-T=1-{\left|\frac{Z-Z_{0}}{Z+Z_{0}}\right|}^{2}=1-{\left|\frac{\sqrt{\mu_{r}}-\sqrt{\varepsilon_{r}}}{\sqrt{\mu_{r}}+\sqrt{\varepsilon_{r}}}\right|}^{2}$$

where *R* represents reflection and *T* represents transmission, *Z*_0_ and *Z* denote the effective impedance of free space and the absorber, respectively. Additionally, $\mu_{r\;}$ and $\varepsilon_{r}\;$ refer to the effective permeability and permittivity of the absorber. Equation (2) provides a theoretical framework to describe how the impedance match between the absorber and free space governs absorption efficiency. It shows that when the effective impedance *Z* of the structure approaches the free-space impedance *Z*_0_, reflection minimizes and absorption approaches unity, assuming negligible transmission due to a thick metallic ground plane. The simplified condition $\varepsilon_{r}$ = $\mu_{r}$ represents an idealized impedance-matching scenario. Experimentally, this condition is rarely satisfied, as most optical materials, including Mxenes, are non-magnetic with *μ_r_* ≈ 1, and exhibit dispersive and lossy permittivity. Metals are essential components in conventional metal–insulator–metal (MIM) designs of metamaterial perfect absorbers (MPAs), typically forming both the top resonant structures and the bottom reflective layers. However, in our design, the top layer is composed of MXene, a 2D material with metallic-like conductivity and plasmonic behavior, which differs from traditional bulk metals. In the long-wave infrared (LWIR) band, the optical response of such materials cannot be fully captured by simple DC surface resistance; hence, we employ the Drude model to describe their permittivity and capture the frequency-dependent plasmonic behavior. The permittivity is expressed as:

(3) $$\varepsilon_{m}=\varepsilon_{1}+i\varepsilon_{2}=\frac{\omega_{p}^{2}}{\omega^{2}+\gamma^{2}}-i\frac{\omega_{p}^{2}\gamma }{\omega \left(\omega^{2}+\gamma^{2}\right)}$$

where *ε*_1_ and *ε*_2_ are the real and imaginary parts of permittivity, *ω_p_* is the plasmon frequency, and *γ* is a damping constant.

## 3. Results and Discussion

### 3.1. Simulation Results

**Figure 2:**
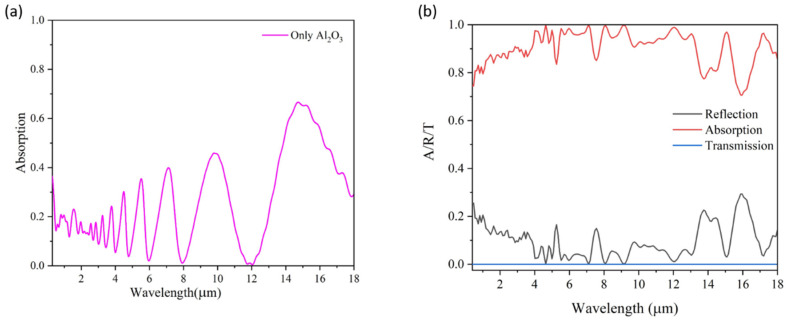
(**a**) Absorption spectrum of only Al_2_O_3_. (**b**) Corresponding absorption, reflection, and transmission spectra of Al_2_O_3_ and Mxene (Ti_3_C_2_O_2_).

A comprehensive analysis of the absorption spectra for the Ti_3_C_2_O_2_ MXene and Al_2_O_3_ layers was conducted over a broad wavelength range from 0.3 μm to 18 μm, as illustrated in Figure 2a,b. Figure 2a illustrates the absorption spectrum of the stand alone Al_2_O_3_ layer, while Figure 2b demonstrates that the transmission remains negligible across the entire wavelength range due to the presence of a thick bottom metallic layer, which effectively blocks light propagation. Concurrently, reflection exhibits significant suppression within the target spectral bands, indicating that the effective impedance of the absorber is closely matched to that of free space. This impedance matching is critical for achieving high absorption. Initially, the Al_2_O_3_ layer alone showed relatively low absorption; however, the addition of the top MXene layer substantially enhanced the absorption efficiency, with peak values exceeding 90%. This improvement underscores the synergistic interaction between the MXene and Al_2_O_3_ layers, which facilitates resonant effects and optimal light trapping, thereby enabling high-performance absorption across the broadband spectral region.

### 3.2. Absorption Mechanism

To support the numerical findings and deepen the physical understanding of the absorber’s behavior, we considered a simplified theoretical model based on effective impedance matching and resonant cavity theory. The observed high absorption is attributed to three main mechanisms: (i) surface plasmon resonance (SPR) induced at the MXene–dielectric interface, (ii) Fabry–Pérot-like resonance formed due to multiple internal reflections between the top and bottom layers, and (iii) impedance matching, which minimizes reflectance by bringing the effective impedance of the structure close to that of free space. While the complete analytical solution is challenging due to the complex, multilayer, and tunable nature of the structure, this simplified model confirms the conditions under which peak absorption occurs. The analytical interpretation of resonance conditions is consistent with FDTD simulation results, validating the design approach.

In the visible to near-infrared (NIR) regime, the dominant absorption mechanism arises from SPRs localized at the MXene–dielectric interface, where the high carrier concentration in Ti_3_C_2_O_2_ facilitates strong confinement and enhancement of the local electromagnetic field. This leads to increased light–matter interaction and high absorptivity in the shorter wavelength region. As the wavelength shifts into the mid-infrared (MIR), Fabry–Pérot resonances become increasingly prominent due to multiple internal reflections within the dielectric spacer (Al_2_O_3_) bounded by the lossy MXene and the metallic substrate, effectively forming a resonant cavity. These standing wave modes trap incident photons, broadening the absorption band. In the long-wave infrared (LWIR) region, instead of relying on magnetic dipole resonances, the structure exhibits impedance matching with free space due to the optimized geometry and material parameters. This reduces reflection at the air–metasurface interface, allowing the incident light to be effectively absorbed even without strong resonant features. The combined contribution of SPRs, FP modes, and impedance matching ensures enhanced and broadband absorption across a wide spectral range.

**Figure 3:**
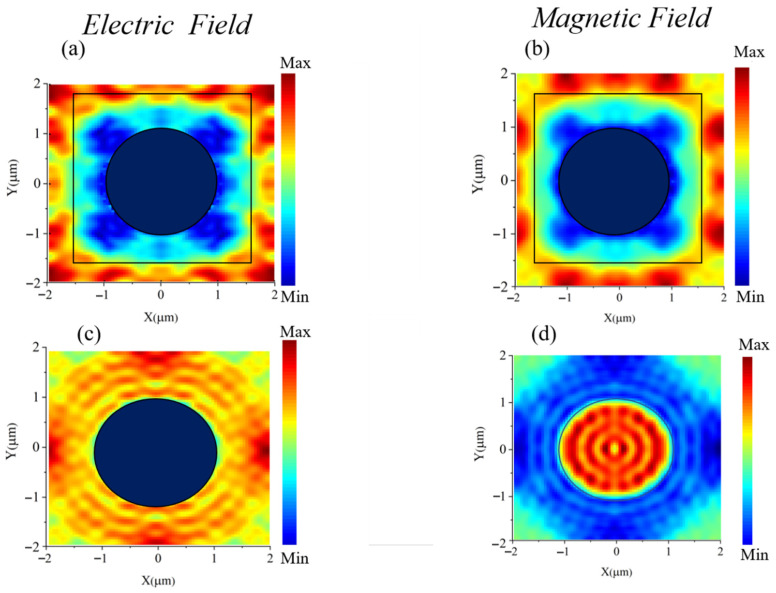
(**a**,**b**) Electric and magnetic field pattern in the x–y plan at the interface of Mxene and Al_2_O_3_; (**c**,**d**) electric and magnetic field pattern in the x–y plan at the upper surface of Mxene.

**Figure 4:**
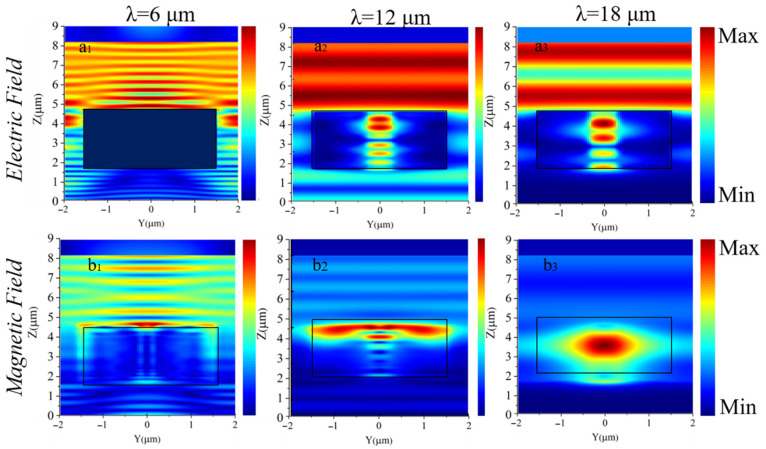
Electric and magnetic field intensity distributions for the metamaterial absorber at different wavelengths. The **top** row (**a_1_**–**a_3_**) displays electric field intensity at varying wavelengths. The **bottom** row (**b_1_**–**b_3_**) illustrates magnetic field intensity at different wavelengths.

The enhanced broadband absorption observed in the MXene-based metamaterial absorber is primarily driven by Fabry–Perot cavity resonances within the MXene-Al_2_O_3_ structure. The dielectric cavity between the MXene layer and the metallic ground plane creates a scenario where incident light undergoes multiple reflections within the cavity, leading to constructive and destructive interference. This interference generates standing wave patterns in both the electric and magnetic fields, which are characteristic of Fabry–Perot resonance. The resonant condition for the Fabry–Perot cavity is satisfied when the cavity thickness *d* aligns with integer multiples of half the wavelength λ, expressed as:

(4) $$d=m\frac{\lambda }{2}$$

where *m* = 1, 2, 3…. This condition leads to constructive interference at specific wavelengths, trapping light within the cavity and increasing the interaction time between the electromagnetic field and the material. The electric and magnetic field patterns observed in Figure 3 and Figure 4 exhibit alternating regions of high and low intensity, consistent with standing waves within a Fabry–Perot cavity. These periodic maxima and minima in field intensity strongly indicate that the absorption enhancement is predominantly due to Fabry–Perot resonance effects rather than solely surface-bound modes.

Furthermore, the high absorption observed in the presence of MXenes, such as Ti_3_C_2_O_2_, is attributed to surface plasmon resonances (SPRs) caused by the metallic nature. These SPRs significantly enhance the light–matter interaction. As incident light strikes the upper surface of the MXene layer, surface plasmon polaritons are excited at the metal-insulator interface and reflected by the underlying metallic layer. This reflection leads to destructive interference, generating resonant modes within the cavity that trap light and prevent transmission through the structure. The integration of Al_2_O_3_ and MXene in the absorber configuration effectively extends the optical path length of light by promoting multiple reflections and scattering inside the structure, hence increasing absorption through broadened interactions with the material.

Additionally, impedance matching between the absorber and free space was found to be a key factor in optimizing absorption efficiency. By adjusting the thickness of the MXene layer and the cavity dimensions, the effective impedance of the absorber is matched to that of free space, which minimizes reflection losses and facilitates near-perfect absorption. This impedance matching is crucial to the device’s performance, as it ensures efficient energy coupling into the absorber with minimal reflection or transmission losses.

Ti_3_C_2_T_x_ MXene, despite its excellent optical and electronic properties, is known to suffer from environmental degradation, especially when exposed to air and humidity. This can lead to oxidation and a gradual decline in performance. However, recent studies have demonstrated that encapsulation using dielectric materials such as Al_2_O_3_ or SiO_2_ can substantially enhance MXene’s environmental stability without significantly affecting its optical response. Since our design already incorporates an Al_2_O_3_ substrate, it can readily support encapsulation layers, thereby improving durability for real-world applications. Al_2_O_3_’s dielectric properties, as reported by Deng et al. [31] for GHz shielding, are also crucial at optical and infrared frequencies. In our absorber, it functions as a dielectric spacer and aids impedance matching, boosting absorption from 0.3 to 18 μm. Future experimental realizations should consider such passivation approaches to ensure long-term functionality.

### 3.3. Electric Magnetic Field Patterns

To identify the dominant absorption mechanisms, we analyzed the electric and magnetic field distributions at various wavelengths shown in Figure 3 and Figure 4. At shorter wavelengths, strong electric field confinement near the MXene surface confirms surface plasmon polariton (SPP) excitation. In the mid-to-long wavelength range, standing wave patterns indicate Fabry–Perot resonances within the dielectric cavity. Additionally, magnetic field localization suggests magnetic dipole modes. These results confirm that the broadband absorption arises from the synergistic effects of SPPs, Fabry–Perot, and magnetic resonances across the spectrum.

A significant factor influencing the observed field patterns is the behavior of electric and magnetic fields within the metamaterial layers. At the interface between the MXene and Al_2_O_3_ layers, mismatches in refractive indices and impedance result in minimum reflection and nearly zero transmission of the waves. Impedance matching minimizes reflection and enhances energy coupling into the structure, enabling Fabry–Pérot resonances that form standing wave patterns characterized by regions of constructive and destructive interference.

As shown in Figure 3a–c, both electric and magnetic fields exhibit minimum intensity at the center and peak near the edges, indicating strong field confinement due to resonant effects. In contrast, Figure 3d shows a magnetic field distribution with maximum intensity at the center and reduced intensity at the edges. This variation reflects the resonant nature of the structure and the orthogonal coupling between electric and magnetic fields, which collectively enhance broadband absorption.

Figure 4(a_1_–b_3_) provides detailed insights into the electric and magnetic field distributions at different wavelengths. These field patterns reveal how the metamaterial absorbs incident electromagnetic waves across the spectrum, offering a deeper understanding of the resonant modes that drive absorption.

The electric field distribution (top row in Figure 4) shows varying confinement patterns at different wavelengths, indicating the excitation of multiple resonant modes within the cavity. At certain wavelengths, the electric field is strongly localized near the MXene surface, suggesting the occurrence of surface plasmon resonance (SPR). At other wavelengths, a more uniform electric field distribution across the cavity reflects Fabry–Perot resonances. In these cases, the optical path length supports constructive interference, which traps light within the cavity and enhances absorption.

The magnetic field patterns (bottom row of Figure 4) reveal complementary resonant behavior, with strong central confinement at specific wavelengths indicative of magnetic dipole resonance. The interaction between electric and magnetic fields at these resonant wavelengths leads to high absorption efficiency, as energy is effectively coupled into the metamaterial structure. The combination of both electric and magnetic field distributions confirms the absorber’s capability to capture and retain electromagnetic energy across a broad wavelength range. Fabry–Perot resonances play a dominant role in enhancing absorption, while surface plasmon polaritons provide additional localized enhancement at the metal–dielectric interface.

This intricate field distribution emphasizes the advanced design of the MXene-Al_2_O_3_ absorber. The optimized material selection and geometric configuration enable broadband absorption, making this structure highly suitable for applications in photo detection, energy harvesting, and electromagnetic shielding.

In the context of real-world applications such as IoT devices, flexible sensors, and wearable electronics, mechanical adaptability is a key consideration. The proposed MXene–Al_2_O_3_-based absorber can potentially be fabricated on flexible substrates such as polyimide or PDMS, due to the ultrathin nature of the functional layers. While our current study focuses on optical and thermal performance, future experimental work will need to address integration challenges such as bending-induced deformation, interfacial adhesion between layers, and scalability of deposition methods. Nonetheless, the inherent chemical stability of Al_2_O_3_ and the 2D flexibility of MXene materials provide a promising foundation for mechanically robust and conformal absorber designs.

## 4. Optimization

The geometric arrangement of the layers, including their thicknesses and interface geometry, plays a crucial role in determining the field distribution patterns. The dimensions of these layers directly influence the wavelengths of light that are efficiently captured and absorbed, which in turn, dictates the resonant modes and localized field improvements. Significant shifts in absorption characteristics are observed when optimizing the MXene layer’s thicknesses, geometry, and radius.

### 4.1. Influence of MXene Layer on Absorption

**Figure 5:**
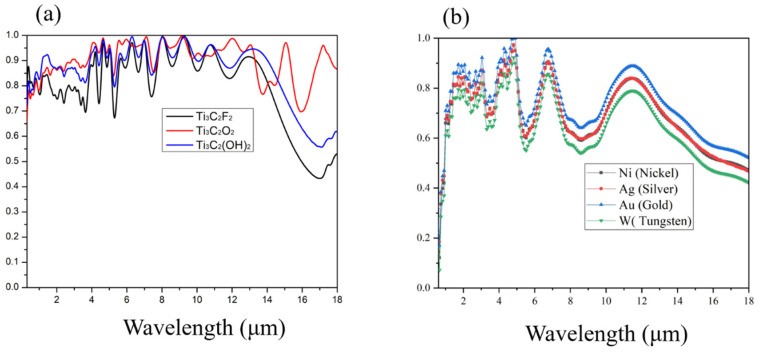
(**a**) The absorption spectra with different MXene layers, (**b**) absorption spectra of the MXene-based metamaterial absorber using Ag, Au, Ni, and W as the bottom metallic layer.

The MXene layer in the metamaterial absorber is key to determining the absorption efficiency and spectral response of the device. Different configurations of MXene, such as Ti_3_C_2_F_2_, Ti_3_C_2_O_2_, and Ti_3_C_2_(OH)_2_, offer distinct electromagnetic properties based on their structural and chemical compositions. As shown in Figure 5a, the absorption spectra of the MXene vary significantly, illustrating the impact of surface termination groups and electronic behavior on light absorption across the 0.3–18 μm wavelength range.

Characterized by its fluorine termination, Ti_3_C_2_F_2_ exhibits exceptional hydrophobic properties, which enhance its stability and durability in harsh environments. This property makes it suitable for electronic devices in challenging conditions or as a corrosion-resistant coating. The fluorine terminations introduce additional absorption channels, significantly improving light-matter interaction and absorption efficiency across a spectrum extending from 0.3 μm to 18 μm.

When characterized by hydroxyl (-OH) functional groups, MXene Ti_3_C_2_(OH)_2_ exhibits hydrophilic characteristics; its flexible nature makes it suitable for energy storage and biomedical applications. The catalytic activity and potential biocompatibility of Ti_3_C_2_(OH)_2_ further expand its application scope.

The surface chemistry of Ti_3_C_2_ MXenes, specifically their surface terminations (O, F, and OH), plays a crucial role in determining their electronic and electromagnetic properties. Ti_3_C_2_O_2_ exhibits high metallic conductivity, which facilitates effective impedance matching to free space and results in strong broadband absorption. In contrast, fluorine-terminated Ti_3_C_2_F_2_ shows enhanced hydrophobicity and stability, contributing additional absorption channels that broaden the spectral response and improve durability under harsh conditions. Hydroxyl-terminated Ti_3_C_2_(OH)_2_ provides increased hydrophilicity and potential biocompatibility but exhibits slightly reduced conductivity, leading to moderately lower absorption efficiency while maintaining broad spectral coverage. Our simulations (Figure 5) confirm these trends and highlight the tunability of MXene optical properties via surface termination engineering, making MXenes highly versatile for tailored absorption applications.”

In Figure 5b, the absorption spectrum exhibits strong absorption in the visible to near-infrared range, followed by a gradual decline in the mid- to far-infrared region. This trend is characteristic of plasmonic metals like silver or gold, which support strong surface plasmon resonances at shorter wavelengths but show reduced absorption at longer wavelengths due to weaker confinement and lower intrinsic losses.

### 4.2. Influence of MXene Shape on Absorption

**Figure 6:**
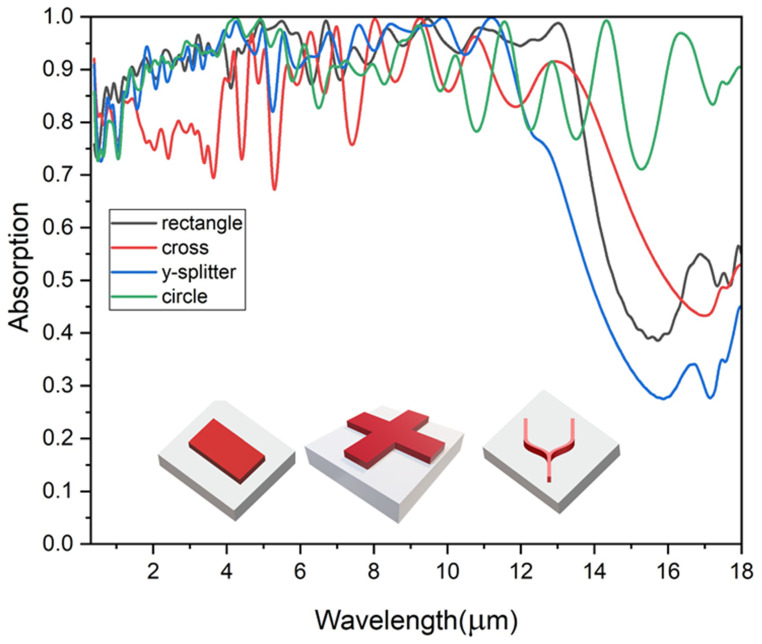
Absorption spectra with different MXene shapes.

In metamaterial design, geometry is crucial in determining absorption properties. The simulations highlight the different absorption behaviors of rectangular, cross, Y splitter, and circular MXene structures, as illustrated in Figure 6. Rectangular, cross, and Y-splitter designs, affected by discontinuities and scattering effects, demonstrate reduced absorption efficiency beyond a critical wavelength due to mode coupling and radiation losses. In contrast, circular structures exhibit stable propagation modes and maintain high absorption efficiency across a broad wavelength range. This consistent performance makes circular geometries ideal for applications requiring broadband absorption.

The impact of MXene shape on resonant absorption is evident through variations in surface plasmon polariton (SPP) excitation and Fabry–Perot cavity effects. Circular MXene shapes support isotropic field distributions, enabling multi-directional SPPs and broad spectral response, enhancing broadband absorption through continuous plasmonic excitation around the perimeter. In contrast, square and rectangular shapes introduce anisotropy, creating longitudinal and transverse resonances that concentrate electric fields at corners, forming localized “hotspots” and discrete absorption peaks. These geometries are more suited to narrowband or multispectral applications. Hexagonal shapes combine these properties, offering both broad absorption and enhanced field localization at vertices, making them ideal for broadband applications.

The electric and magnetic field distributions observed in various MXene shapes demonstrate how geometry influences resonant behavior. Circular shapes generate radially symmetric fields conducive to multi-wavelength SPPs excitation, while square and rectangular shapes yield enhanced field confinement at edges, suggesting Fabry–Perot resonance and standing waves within the cavity. The circular MXene geometry exhibits superior broadband absorption due to its radial symmetry, which supports isotropic resonant behavior. Unlike rectangular or elliptical shapes that exhibit direction-dependent responses, the circular shape enables uniform electric and magnetic field confinement regardless of the polarization or incident angle of the incoming light. This results in consistent resonance coupling across a wider range of wavelengths and directions. Such isotropic absorption characteristics are particularly advantageous for omnidirectional light harvesting and photodetection applications, where consistent performance under varying illumination angles is critical. As seen in Figure 6, rectangle, cross, and Y-shaped MXene patterns show reduced absorption at longer wavelengths due to geometric limitations. Sharp edges cause radiation losses and poor plasmon coupling, weakening resonance. In contrast, circular shapes maintain isotropic field confinement, ensuring strong broadband absorption.

### 4.3. Influence of the Radius of MXene on Absorption

**Figure 7:**
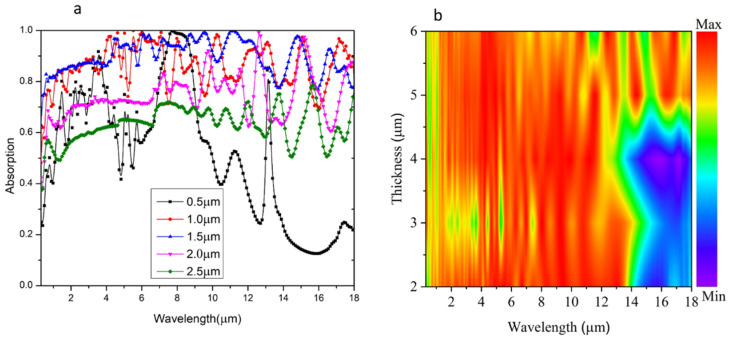
(**a**) Absorption spectra at different Mxene radii (**b**) Absorption spectra at different thickness of Mxene.

The diameter of disk or pillar arrays within the MXene-based metamaterial significantly impacts absorption characteristics. Our simulations show how varying the radius of a disk or pillar from 0.5 μm to 2.5 μm affects absorption spectra, as shown in Figure 7a.

When the MXene radius is small, the electric field concentrates near the center, creating localized “hotspots” where light-matter interaction is maximized. This results in sharper, more defined absorption peaks. In contrast, a larger MXene radius allows the electric field to spread over a wider area, supporting a broader range of resonant modes. This extended field distribution is advantageous for broadband absorption, making it ideal for applications such as solar energy harvesting.

Additionally, changes in the MXene radius impact both surface plasmon and Fabry–Perot resonance conditions within the absorber. A larger radius supports more extensive surface plasmonic modes across the MXene-dielectric interface, allowing for broader coupling of light across different wavelengths. Simultaneously, a larger radius extends the optical path length within the cavity, which can shift Fabry–Perot resonance peaks and trap light more effectively across a wider spectral range. This combination of expanded plasmonic and Fabry–Perot resonances allows a larger MXene radius to achieve broadband absorption, making it ideal for applications like solar energy harvesting. Conversely, a smaller radius, with more focused resonant peaks, is well-suited for wavelength-selective applications such as multispectral sensing, where specific wavelengths are targeted for absorption.

Figure 7b illustrates the absorption spectra as a function of MXene thickness using a heat map representation. The results reveal a clear thickness-dependent absorption behavior: as the thickness of the MXene layer decreases, the absorption becomes more pronounced at shorter wavelengths (visible to the near-infrared range), while it gradually diminishes in the mid-to-long infrared region. This trend can be attributed to the reduced optical path length and decreased interaction volume for incident light at lower thicknesses, which favors the excitation of localized surface plasmons and enhances resonance at shorter wavelengths. Conversely, thicker MXene layers support stronger multiple internal reflections and cavity effects, facilitating improved light trapping at longer wavelengths. Thus, the MXene thickness plays a critical role in tailoring the spectral position and efficiency of absorption across the broadband range, which shows that increasing the MXene thickness enhances absorption in the short-wavelength (visible) range due to stronger field confinement. However, this can slightly narrow the absorption bandwidth in the long-wavelength (mid-IR) region, primarily due to increased optical path losses and impedance mismatch resulting from a reduced skin depth.

Controlled simulations with ±10% variation in MXene geometry showed <5% fluctuation in average absorption, confirming design robustness.

### 4.4. Influence of Structure of MXene on Absorption

**Figure 8:**
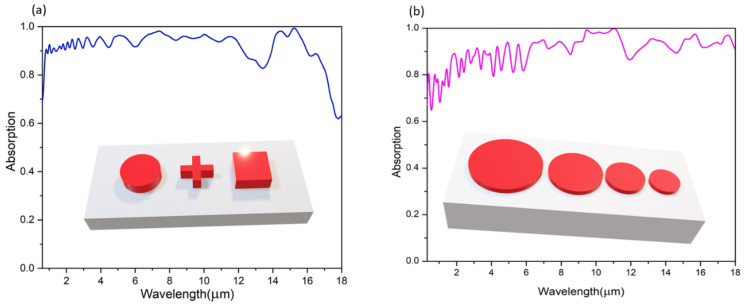
(**a**) Absorption spectrum for Supercell I. (**b**) Absorption spectrum for Supercell II.

The study of MXene-based nanostructures, comprising circles, rectangles, and crosses, based on a 1 μm thick Al_2_O_3_ layer, reveals notable spectral absorption properties across a wide wavelength range. As illustrated in Figure 8a, these structures exhibit an absorption efficiency of 90% from 0.3 μm to 13 μm. This broad-spectrum efficiency is due to several factors inherent to MXene materials. Firstly, MXenes possess a complex electronic band structure with a high density of states near the Fermi level, facilitating efficient photon absorption across various wavelengths, including the visible and infrared light. Additionally, the engineered MXene nanostructures, tailored to match the incident light wavelength, promote the excitation of surface plasmon resonances (SPRs), which enhance electromagnetic field confinement and absorption. Moreover, the geometry of the MXene nanostructures, in conjunction with the Al_2_O_3_ substrate, causes multiple reflections and scattering of incident photons, further improving absorption by increasing the optical path length within the material. Interestingly, the absorption efficiency peaks at 95% within the 14 μm to 16 μm range, suggesting additional absorption mechanisms or resonance phenomena at longer wavelengths. These findings underscore the versatility and tunability of MXene-based nanostructures, making them adaptable for various photonics, sensing, and thermal management applications.

Further simulations, shown in Figure 8b, explore the effect of varying disk radii (0.5 μm to 2 μm) at a periodicity of 5 μm. Larger disks result in increased average absorption, as they provide a greater surface area for photon interactions. Larger disks also facilitate better light trapping within the MXene-based nanostructures, prolonging the optical path length and enhancing photon-matter interactions through multiple reflections and scattering. Additionally, larger disks enhance resonant coupling with incident light, boosting absorption in specific spectral regions. This increase in localized field enhancement and confinement of incident light further optimizes photon absorption.

This comprehensive analysis underscores the critical role of structural design in optimizing the optical properties of MXene-based metamaterials. By carefully tuning the geometry and size of the constituent elements, we can significantly improve the performance of these structures for a variety of optoelectronic and photonic applications. MXene-based absorbers, while promising for broadband light harvesting, can be sensitive to temperature-induced phonon scattering, which may degrade their performance in high-temperature environments. To overcome this limitation, recent studies have shown that introducing cationic disorder and dimensional constriction in high-entropy MXenes can significantly suppress lattice thermal conductivity by enhancing phonon scattering and reducing phonon mean free paths. This approach can be leveraged during fabrication to improve the thermal resilience of MXene-based absorbers, ensuring stable and efficient operation under practical energy harvesting conditions.

While this study primarily focuses on simulated electromagnetic performance, we acknowledge the importance of thermal and mechanical stability for practical applications. Ti_3_C_2_O_2_ MXenes demonstrate good thermal stability up to moderate temperatures (~300–400 °C) and exhibit mechanical flexibility due to their layered two-dimensional structure, which compares favorably to conventional carbon-based and polymer composite absorbers. Moreover, recent advances in material engineering, such as introducing cationic disorder and dimensional constriction, have shown promise in enhancing phonon scattering and suppressing lattice thermal conductivity, thereby improving thermal resilience [32]. These properties suggest that MXene-based absorbers can maintain performance under realistic environmental stresses, including heat, humidity, and mechanical deformation. Experimental validation of these stability aspects is underway and will be addressed in future work.

**Figure 9:**
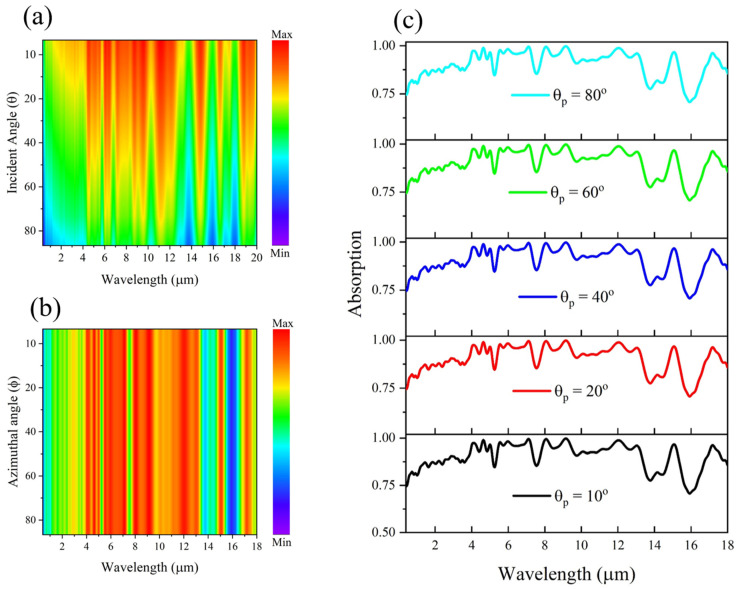
Angular and polarization dependence of the absorption performance of the MXene-based metamaterial absorber: (**a**) Heat map showing absorption as a function of incident angle, demonstrating high absorption up to 70°; (**b**) heat map of absorption spectra at various azimuthal angles; (**c**) absorption spectra under different polarization angles. The absorber exhibits strong angular and polarization insensitivity due to its symmetric structure and robust resonant mechanisms.

Figure 9a–c illustrates the angular stability of the proposed MXene-based metamaterial absorber. As shown in Figure 9a, the heat map of absorption versus incident angle demonstrates that the absorber maintains high absorption up to an incident angle of 70°, with only a slight reduction beyond this point. This robustness can be attributed to the strong confinement of the electromagnetic fields within the MXene–Al_2_O_3_ cavity, which minimizes angular sensitivity. Figure 9b,c shows the absorption performance under varying azimuthal and polarization angles, respectively. In both cases, the absorption remains nearly invariant, indicating polarization-insensitive behavior. This angular and polarization stability arises from the symmetric structural design and the excitation of localized resonances such as surface plasmon polaritons (SPPs) and Fabry–Pérot modes, which are less dependent on the angle or polarization of incident light. Such characteristics are crucial for real-world applications where light may impinge from diverse directions.

In Table 1 and Table 2 the results highlight that MXene-based absorbers (this work) achieve ultra-broadband absorption from 0.3 to 18 µm with >95% peak absorption using a simple, bias-free planar design. In contrast:

- Graphene–metal hybrids generally offer high tunability and flexibility but often require external doping or biasing and typically show narrower operational bands or reduced peak absorption.
- Transition-metal oxide (TMO) structures exhibit good thermal stability and moderate flexibility but involve more complex multilayer fabrication.
- Metal–dielectric–metal (MDM) designs achieve high peak absorption but suffer from bulkiness and lower mechanical flexibility.
- Carbon nanotube (CNT)-based absorbers show broadband response and scalability, but their disordered nature can lead to less predictable performance.

## 5. Conclusions

The proposed high-efficiency broadband absorber, consisting of a Ti_3_C_2_T_x_ layer and Al_2_O_3_ substrate, operates effectively across the visible and near-infrared regions. Through optimization of the absorber’s structural parameters, it achieves an average absorption of over 85–90% across the 300 nm to 18,000 nm wavelength range. Theoretical analysis using the FDTD method indicates that the enhanced absorption and broadband response are primarily attributed to the localized surface plasmon resonance (LSPR) of MXene nanoparticles and the Fabry–Perot (FP) resonance between the MXene and Al_2_O_3_ layers. The performance of this absorber exceeds that of most currently reported materials, offering promising potential for applications in solar energy harvesting, solar water evaporation, and related fields.

## Figures and Tables

**Table 1:** Comparison of the absorber presented in this paper with previously published broadband absorbers in the visible and near-infrared regions.

Reference	Main Materials	Absorption Bandwidth	Absorption Efficiency	Fabrication Methode	Flexibility
[33]	Mxene and Au	500–1500 nm	about 85%	Sputtering and spin-coating	no
[34]	Mxene, tungsten	500–2500 nm	90%	Coating and self-assembly	yes
[35]	MXene, Au, and Al_2_O_3_	600–2100 nm	80–90%	Layer-by-layer deposition	no
[36]	Mxene, SiO_2_	400–2500 nm	85%	Spin-coating	yes
This paper	Mxene, Al_2_O_3_	300–18,000 nm	85–90%	Spin-coating and deposition	yes

**Table 2:** Comparison of broadband absorbers based on material type.

Reference	Absorber Type	Spectral Range (µm)	Peak Absorption (%)	Structural Complexity	Flexibility
This work	MXene (Ti_3_C_2_T_x_) (This Work)	0.3–18	>95	Simple, planar, no bias	Flexibility
[37]	Graphene–Metal Hybrid	~2–10	~85–95 (narrowband)	Requires doping	Moderate
[38]	Transition Metal Oxides (TMOs)	~0.5–12	~80–95	Multilayer, high-temp stable	High
[39]	Metal–Dielectric–Metal (MDM)	~1–15	~90–98	Resonator-based, bulky	Low
[40]	Carbon Nanotube-Based	~0.8–1.6	~95–98 (narrow-band)	Disordered layer, scalable	Moderate

## Data Availability

The raw data supporting the conclusions of this article will be made available by the authors on request.

## References

[1] Huang M., Wang L., Pei K., Li B., You W., Yang L., Zhou G., Zhang J., Liang C., Che R. (2024). Heterogeneous Interface Engineering of Bi-Metal MOFs-derived ZnFe_2_O_4_–ZnO-Fe@C Microspheres via Confined Growth Strategy Toward Superior Electromagnetic Wave Absorption. Adv. Funct. Mater..

[2] Huang X., Qiao M., Lu X., Li Y., Ma Y., Kang B., Quan B., Ji G. (2021). Evolution of dielectric loss-dominated electromagnetic patterns in magnetic absorbers for enhanced microwave absorption performances. Nano Res..

[3] Xiao J., He M., Zhan B., Guo H., Yang J., Zhang Y., Qi X., Gu J. (2024). Multifunctional microwave absorption materials: Construction strategies and functional applications. Mater. Horiz..

[4] Zhang X., Tian X.L., Qin Y., Qiao J., Pan F., Wu N., Wang C., Zhao S., Liu W., Cui J. (2023). Conductive metal–organic frameworks with tunable dielectric properties for boosting electromagnetic wave absorption. ACS Nano.

[5] Chen Y., Qiang R., Shao Y., Yang X., Ma Q., Xue R., Chen B., Feng S., Ren F., Ding Y. (2024). Fe3C/Fe implanted hierarchical porous carbon foams for lightweight and broadband microwave absorption. Diam. Relat. Mater..

[6] Yu M., Li S., Ren X., Liu N., Guo W., Xue J., Tan L., Feng S., Ren F., Ding Y. (2024). Magnetic bimetallic heterointerface nanomissiles with enhanced microwave absorption for microwave thermal/dynamics therapy of breast cancer. ACS Nano.

[7] Song Q., Ye F., Kong L., Shen Q., Han L., Feng L., Yu G., Pan Y., Li H. (2020). Graphene and MXene nanomaterials: Toward high-performance electromagnetic wave absorption in gigahertz band range. Adv. Funct. Mater..

[8] Liu Z., Wang K., Zhang H., Huang S., Xie N., Wang Q., Zhu H., Liu Y., Zhao X. (2024). Solvothermally Synthesized Co (CoO)/Ti_3_C_2_T_x_/TiO_2_ Nanocomposites for Enhanced Microwave Absorption. ACS Appl. Nano Mater..

[9] Mehmood M.Q., Shah A.R., Naveed M.A., Mahmood N., Zubair M., Massoud Y. (2023). MXene-based polarization-insensitive UV-VIS-NIR meta-absorber. IEEE Access.

[10] Berdiyorov G.R. (2016). Optical properties of functionalized Ti_3_C_2_T_2_ (T = F, O, OH) MXene: First-principles calculations. AIP Adv..

[11] Akhter R., Maktedar S.S. (2023). MXenes: A comprehensive review of synthesis, properties, and progress in supercapacitor applications. J. Mater..

[12] Navitski I., Ramanaviciute A., Ramanavicius S., Pogorielov M., Ramanavicius A. (2024). MXene-based chemo-sensors and other sensing devices. Nanomaterials.

[13] Li Y., Xiong C., Huang H., Peng X., Mei D., Li M., Liu G., Wu M., Zhao T., Huang B. (2021). 2D Ti_3_C_2_T_x_ MXenes: Visible black but infrared white materials. Adv. Mater..

[14] Li X., Yin X., Han M., Song C., Xu H., Hou Z., Zhang L., Cheng L. (2017). Ti_3_C_2_ MXenes modified with in situ grown carbon nanotubes for enhanced electromagnetic wave absorption properties. J. Mater. Chem. C.

[15] Valipour A., Kargozarfard M.H., Rakhshi M., Yaghootian A., Sedighi H.M. (2022). Metamaterials and their applications: An overview. Proc. Inst. Mech. Eng. Part L J. Mater. Des. Appl..

[16] Hou S.G., Liang L., Deng S.H., Chen J.F., Huang Q., Cheng Y., Fan C.H. (2014). Nanoprobes for super-resolution fluorescence imaging at the nanoscale. Sci. China Chem..

[17] Kim K., Jang K.-W., Bae S.-I., Kim H.-K., Cha Y., Ryu J.-K., Jo Y.-J., Jeong K.-H. (2021). Ultrathin arrayed camera for high-contrast near-infrared imaging. Opt. Express.

[18] Xu Y., Bai P., Zhou X., Akimov Y., Png C.E., Ang L.-K., Knoll W., Wu L. (2019). Optical refractive index sensors with plasmonic and photonic structures: Promising and inconvenient truth. Adv. Opt. Mater..

[19] Camayd-Muñoz P., Ballew C., Roberts G., Faraon A. (2020). Multifunctional volumetric meta-optics for color and polarization image sensors. Optica.

[20] Wang D., Liu Z., Wang H., Li M., Guo J.L., Zhang C. (2023). Structural color generation: From layered thin films to optical metasurfaces. Nanophotonics.

[21] Asano T., Suemitsu M., Hashimoto K., De Zoysa M., Shibahara T., Tsutsumi T., Noda S. (2016). Near-infrared–to–visible highly selective thermal emitters based on an intrinsic semiconductor. Sci. Adv..

[22] Rinnerbauer V., Lenert A., Bierman D.M., Yeng Y.X., Chan W.R., Geil R.D., Senkevich J.J., Joannopoulos J.D., Wang E.N., Soljačić M. (2014). Metallic photonic crystal absorber-emitter for efficient spectral control in high-temperature solar thermophotovoltaics. Adv. Energy Mater..

[23] Qi B., Shou H., Zhang J., Chen W., Feng J., Niu T., Mei Z. (2023). A near-perfect metamaterial selective absorber for high-efficiency solar photothermal conversion. Int. J. Therm. Sci..

[24] Park S., Heo S.W., Lee W., Inoue D., Jiang Z., Yu K., Jinno H., Hashizume D., Sekino M., Yokota T. (2018). Self-powered ultra-flexible electronics via nano-grating-patterned organic photovoltaics. Nature.

[25] Chang C.-C., Kort-Kamp W.J.M., Nogan J., Luk T.S., Azad A.K., Taylor A.J., Dalvit D.A.R., Sykora M., Chen H.-T. (2018). High-temperature refractory metasurfaces for solar thermophotovoltaic energy harvesting. Nano Lett..

[26] Stewart J.W., Vella J.H., Li W., Fan S., Mikkelsen M.H. (2020). Ultrafast pyroelectric photodetection with on-chip spectral filters. Nat. Mater..

[27] Frydendahl C., Grajower M., Bar-David J., Zektzer R., Mazurski N., Shappir J., Levy U. (2020). Giant enhancement of silicon plasmonic shortwave infrared photodetection using nanoscale self-organized metallic films. Optica.

[28] Naguib M., Mashtalir O., Carle J., Presser V., Lu J., Hultman L., Gogotsi Y., Barsoum M.W. (2012). Two-dimensional transition metal carbides. ACS Nano.

[29] He P., Wang X.-X., Cai Y.-Z., Shu J.-C., Zhao Q.-L., Yuan J., Cao M.-S. (2019). Tailoring Ti_3_C_2_T_x_ nanosheets to tune local conductive network as an environmentally friendly material for highly efficient electromagnetic interference shielding. Nanoscale.

[30] Luo H., Feng W., Liao C., Deng L., Liu S., Zhang H., Xiao P. (2018). Peaked dielectric responses in Ti_3_C_2_ MXene nanosheets enabled composites with efficient microwave absorption. J. Appl. Phys..

[31] Gashti M.P., Eslami S. (2012). Structural, optical and electromagnetic properties of aluminum–clay nanocomposites. Superlattices Microstruct..

[32] Sharma P., Singh P., Balasubramanian G. (2024). Engineering phonon transport through cation disorder in dimensionally constricted high entropy MXene. Carbon.

[33] Chaudhuri K., Alhabeb M., Alhabeb M., Wang Z., ShalaevYury V.M., Boltasseva G. (2018). Highly Broadband Absorber Using Plasmonic Titanium Carbide (MXene). ACS Photonics.

[34] Jia Y., Wu T., Wang G., Jiang J., Miao F., Gao Y. (2022). Visible and Near-Infrared Broadband Absorber Based on Ti_3_C_2_T_x_ MXene. Nanomaterials.

[35] Jiang Y., Deng J., Lu Y., Xie Z., Chen Y. (2024). Large-scale, transferable and double-sided metasurface absorber for the visible to near-infrared spectrum. Surf. Interfaces.

[36] Li L., Ren Y., Cui W., Wang Y., Yang Z., Wu X., Huo Y., Li G., Zhao Y., He Z. (2024). High efficiency ultra-broadband absorber and thermal emitter for the composite Ag-NPs/SiO_2_/MXene multilayer structure on ITO substrate. Opt. Laser Technol..

[37] Yan H., Li X., Chandra B., Tulevski G., Wu Y., Freitag M., Zhu W., Avouris P. (2012). Tunable infrared plasmonic devices using graphene/insulator stacks. Nat. Nanotechnol..

[38] Thongrattanasiri S., Koppens F.H.L., de Abajo F.J.G. (2012). Complete optical absorption in periodically patterned graphene. Phys. Rev. Lett..

[39] Landy N., Sajuyigbe S., Mock J.J., Smith D.R., Padilla W.J. (2008). Perfect metamaterial absorber. Phys. Rev. Lett..

[40] Obraztsov P.A., Sirotkin A.A., Obraztsova E.D., Svirko Y.P., Garnov S.V. (2010). Carbon-nanotube-based saturable absorbers for near infrared solid state lasers. Opt. Rev..

